# Description of the Molecular and Phenotypic Spectrum of Lesch-Nyhan Disease in Eight Chinese Patients

**DOI:** 10.3389/fgene.2022.868942

**Published:** 2022-04-26

**Authors:** Lu Li, Xiaohui Qiao, Fei Liu, Jingjing Wang, Huijun Shen, Haidong Fu, Jian-Hua Mao

**Affiliations:** ^1^ Department of Nephrology, Children’s Hospital, National Clinical Research Center for Child Health, National Children’s Regional Medical Center, Zhejiang University School of Medicine, Hangzhou, China; ^2^ Department of Nephrology, Ningbo Women and Children’s Hospital, Ningbo, China

**Keywords:** Lesch-Nyhan disease, HPRT1 gene, self-mutilation, hyperuricemia, dystonia

## Abstract

**Background:** Lesch-Nyhan disease (LND) is a rare disorder involving pathogenic variants in the *HPRT1* gene encoding the enzyme hypoxanthine-guanine phosphoribosyltransferase (HGPRT) that result in hyperuricemia, intellectual disability, dystonic movement disorder, and compulsive self-mutilation. The purpose of the present study was to characterize the genetic basis of LND and describe its phenotypic heterogeneity by identifying the variation in the *HPRT1* gene in a cohort of Chinese LND patients.

**Results:** The median age at diagnosis was 31 mo (interquartile range (IQR): 7–76 mo), and the initial manifestations were mainly head control weakness and motor development delay. The median age of self-mutilation behavior onset was 19 mo (IQR: 17–24 mo), and all patients were required to travel in a wheelchair and fall into the predicament of compulsive self-harm behavior. There were two patients whose blood uric acid levels were normal for their high urinary acid excretion fraction without taking uric acid-lowering drugs. Seven different pathogenic variants of the *HPRT1* gene were identified among eight independent pedigrees, including four novel mutations [c.299 (exon 3) T > A; loss (exon: 6) 84 bp; c.277_281delATTGC; c.468_470delGAT]. The pathogenic variant sites were mainly concentrated in exon 3, and truncating mutations (including frameshift mutations and nonsense mutations) were the most common genetic variant types (5/7, 71.4%).

**Conclusion:** The present study described the phenotypic and molecular spectrum of LND in eight Chinese families, including four novel mutations, which expands our understanding of LND.

## Introduction

Lesch-Nyhan disease (LND: OMIM 3000322) is a rare neurogenetic disorder involving pathogenic variants in the *HPRT1* gene encoding the enzyme hypoxanthine-guanine phosphoribosyltransferase (HGPRT). The *HPRT1* gene spans ∼44 kb of DNA at Xq26.2-q26.3, including 8 introns and 9 exons, encoding a total of 218 amino acids with a protein size of 24.5 kDa (Torres and Puig, 2007; Fu et al., 2014). To date, more than 600 pathogenic variants associated with LND have been identified (Nguyen et al., 2017). Deficiency of the enzyme HGPRT is usually associated with clinical evidence for overproduction of uric acid and a series of neurobehavioral problems. Patients with enzyme HGPRT activity less than 2% often have characteristic self-injurious behaviors (buccal mucosa biting, lip biting, tongue and finger biting), dystonic movement disorder, intellectual disability and hyperuricemia, namely, LND (Jinnah). The prevalence of LND is approximately 1/380,000 live births in Canada (Crawhall et al., 1972) and 1/235,000 live births in Spain (Roche et al., 2009). The life expectancy of LND patients can reach 20–40 years old under effective clinical management (Jinnah). Aspiration pneumonia and renal failure are the main causes of death (Fasullo and Endres, 2015). Patients with LND are troubled and hurt by self-injurious behaviour (SIB) (Schretlen et al., 2005). Until now, there has been no effective drug that can control SIBs, as the pathophysiology between SIBs and HGPRT deficiency is not clear (Seifert, 2016). Although reports about LND are not uncommon around the world (Puig et al., 2001; Schretlen et al., 2013; Cho et al., 2019), there are only a few single-case reports about LND in Chinese patients (Huang et al., 2018; Jian* et al., 2013; Lee et al., 1995). The present study aims to describe Chinese patients with LND with the purpose of improving knowledge of the natural history of the disease and outlining the background for future management recommendations.

## Materials and Methods

### Patients and Ethical Approval

This study was approved by the Ethics Committee of Children’s Hospital of Zhejiang University and followed the Declaration of Helsinki. Informed consent was obtained from the parents of the patients. A total of eight pediatric patients from eight unrelated families diagnosed with LND in the Children’s Hospital of Zhejiang University School of Medicine from May 2018 to August 2021 were included in this study. LND was diagnosed according to recognized criteria: evidence of excess uric acid production, characteristic neurobehavioral phenotype, a pathogenic variant in *HPRT1* identified by molecular genetic testing and/or HGPRT enzyme activity <2% (Jinnah; Jinnah et al., 2006). Clinical features such as hyperuricemia, motor dysfunction, and SIB were retrospectively reviewed from the medical records. The assessment of the patient’s renal function followed the updated Schwartz (CKiDCr) eGFR = 0.413 × L (cm)/PCr (mg/dl) (Schwartz et al., 2009).

### Targeted Next-Generation Sequencing and Data Analysis

Genomic DNA was extracted from peripheral blood, and its integrity was assessed by 0.8% agarose gel electrophoresis. The whole-exome library was constructed by a Roche Nimble Gen SEQ EZ exome enrichment kit v2.0 and seq EZ exome enrichment kit v2.0 according to industrial instructions. The samples were sequenced using the Illumina NovaSeq 6,000 series sequencer (PE150) according to the standard manual. After deleting adapters, low-quality read filters, and other quality control protocols, the raw data were cleaned up. The clean data were aligned with the NCBI human reference genome (hg18) using Burrows Wheeler Aligner (BWA), and variants were called using Genome Analysis Toolkit (GATK). Samtools and Pindel were used to call single nucleotide polymorphisms (SNPs) and InDels (insertion-deletions), respectively. The clean data were then filtered according to the quality of the sequencing for further analysis. Nonsynonymous substitutions and SNPs with minor allele frequencies (MAFs) lower than 5% were filtered using Scale-invariant feature transform (SIFT). Then, the function and pathogenicity of the mutant gene were analysed referencing the dbSNP (http://www3.ncbi.nlm.nih.gov/SNP/), 1000 Genomes Project (ftp://ftp-trace.ncbi.nih.gov/1000genomes/), ExAC (https://exac.broadinstitute.org/), ESP (https://evs.gs.washington.edu/EVS/), OMIM (ncbi.nlm.nih.gov/omim/), Swiss-Var (http://swissvar.expasy.org), HGMD (http://www.hgmd.org), ClinVar (https://www.ncbi.nlm.nih.gov/clinvar/), and other disease databases. Protein structure prediction software, such as PROVEAN (http://provean.jcvi.org/index.php), SIFT (http://sift.jcvi.org/), PolyPhen-2 (http://genetics.bwh.harvard.edu/pph2/), and Mutationtaster (http://mutationtaster.org), was used to screen variants with unknown single base pathogenicity. MaxEntScan (http://genes.mit.edu/burgelab/maxent/Xmaxentscan_scoreseq.html) was used to screen potential splice sites. The classification and pathogenicity of variants were conducted according to the variant-interpretation guidelines from the American College of Medical Genetics and Genomics (AGMG) (Richards et al., 2015).

## Results

### Clinical Characteristics

#### Overview

Table 1 summarizes the clinical characteristics of eight patients with LND when they were diagnosed in the present study. The median age of self-mutilation behavior onset was 19 mo (interquartile range (IQR): 17–24 mo), and the median age of diagnosis was 31 mo (IQR: 7–76 mo). Two patients were diagnosed by gene testing before the appearance of SIB. All eight patients presented baseline hypotonia, severe action dystonia and compulsive SIB. SIB mainly manifests as biting lips, buccal mucosa, tongue, and fingers. No patient showed any aggressive behavior towards other people or objects. One patient observed a seizure. There was no obvious abnormality in brain magnetic resonance imaging (MRI), except for some cases with a widening of the extracerebral space or dysmyelination. All eight patients had varying degrees of eating disorders (chewing and swallowing dysfunction). Megaloblastic anemia was observed in one patient. Hyperuricaemia manifested in seven patients, and two patients maintained normal blood uric acid without taking urate-lowering drugs. Three patients were found to have kidney stones or crystals at the time of diagnosis, one patient had nephrocalcinosis, and one patient showed an unclear boundary between the renal cortex and medulla at the time of diagnosis. Two patients showed varying degrees of decline in renal function, one of which was complicated with microalbuminuria. Sleep disorders (difficulty falling asleep and/or frequent waking up) plagued every patient. Seven patients needed body restraints (such as elbow restraints, gloves, and bandages) to prevent self-injury. None of the eight patients had their teeth extracted. Some patients (patients 1, 2, 4, and 6) were misdiagnosed with “cerebral palsy” before the diagnosis of LND, and patient 7 was misdiagnosed with “global developmental delay” before the symptoms of SIB.

**TABLE 1:** Clinical features of eight Chinese patients from eight unrelated families with LND when they are diagnosed.

Patients	P1	P2	P3	P4	P5	P6	P7	P8
Sex	M	M	M	M	M	M	M	M
Family history	–	–	–	+	–	–	–	–
Age at presentation	5 m	4 m	4 m	3 m	4 m	3 m	4 m	5 m
Age of diagnosis	151 m	42 m	6 m	76 m	7 m	96 m	20 m	12 m
Age of self-mutilation onset	36 m	17 m	14 m	36 m	21 m	24 m	17 m	17 m
Baseline hypotonia	+	+	+	+	+	+	+	+
Twisting	+	+	–	+	+	–	+	–
Spasms	+	+	+	+	+	+	+	+
Developmental delay	+	+	+	+	+	+	+	+
Self-injurious behavior	+	+	+	+	+	+	+	+
Brain MRI	Nor	ESW	Nor	Nor	ESW	ESW	Nor	DML
Epilepsy	+	–	–	–	–	–	–	–
^a^Serm uric acid (umol/L)	439	671	677	592	924	327	378	569
Renal ultrasonography	KS	UBBCM	NPC	NA	KS	Nor	Nor	KC
Blood creatinine (umol/L)	65	66	18	38	19	39	25	24
eGFR(ml/min/1.73 m^2)	68.5	53	117	103	115	112	102	106
Proteinuria	–	+	–	–	–	–	–	–
Hematuria	–	–	–	–	–	–	–	–
Gouty arthritis	–	–	–	–	–	–	–	–
Megaloblastic anemia	–	+	–	–	–	–	–	–
Eating disorder	+	+	+	+	+	+	+	+
Sleep disorder	+	+	+	+	+	+	+	+
Body restraints	+	+	+	+	+	+	+	–
Dental pads	–	–	–	–	–	–	–	–

a Serm uric acid (µmol/L): The normal range for the plasma uric acid in male children refers to the previous literature reported^19^: <5 years 214 ± 53.6; 5–10 years 244 ± 59.5; 10–12 years 262 ± 65.5; 12–15 years 333 ± 65.5.

DML, dysmyelination; ESW, extracerebral space widened; KC, Kidney Crystal; KS, Kidney stones; M, male; Nor, normal; NPC, nephrocalcinosis; UBBCM, unclear boundary between the cortex and the medulla; eGFR, estimated glomerular filtration rate, following the updated Schwartz (CKiDCr) eGFR = 0.413 × L (cm)/PCr (mg/dl)^17^.

### Developmental Delay Characteristics

All eight patients denied a history of perinatal trauma and showed abnormal motor development with poor head control at the age of 3–5 months. All patients were never able to sit alone, crawl or walk and relied on a wheelchair to get around. Two patients (patient 2 and patient 8) showed regression of motor development because they were able to control their heads at the age of 3 months. All patients underwent rehabilitation for at least 6 months to 2 years, but the effect was poor.

### Self-Mutilation Behavior Characteristics

**FIGURE 1:**
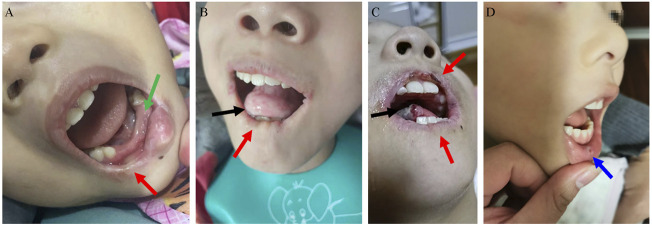
Self-mutilation symptoms in LND. **(A)**: Patient 2’s right lower lip became thinner (red arrow) due to his bite, and the lower central and lateral incisors were also worn away by himself (green arrow). **(B)**: Patient 1’s lower lip became thinner (red arrow), and the tongue became shorter due to long-term self-injurious behaviour (SIB). **(C)**: Patient 6’s lip became mutilated due to SIB (red arrow), and the tip of his tongue was often battered by his bite (black arrow). **(D)**: Bitting of the mucosa in patient 8 (blue arrow).

Most patients have shocking and cruel SIB, mainly manifesting as biting of the lips, buccal mucosa, tongue, and fingers. The median age of SIB onset was 19 mo (IQR: 17–24 mo). S-adenosylmethionine (SAMe) (23 mg/kg.d) was administered to patient 8 for more than 3 months, while symptoms of self-injury did not improve. SIB can be aggravated when patients are nervous, unfamiliar, and sick. Often, muscle tone increases when they feel nervous and restless. As shown in Figure 1: Patient 1’s lower lip became thinner and the tongue became shorter due to long-term SIB; patient 2’s right lower lip became thinner due to his bite, and the lower central and lateral incisors were also worn away by himself; patient 6’s lip became mutilated due to SIB, and the tip of his tongue was often battered by his bite; Patient 8’s self-mutilation behavior was the least among all patients, and he likes to bite the oral mucosa. The parents of patients 1 and 6 reported that children with LND felt anxious and scared when the protective restraint device was removed or not properly applied.

### Characteristics of Hyperuricaemia

Six patients presented with hyperuricemia at the time of diagnosis, with an average uric acid level of 580.5 μmol/L (IQR: 378–671 μmol/L). However, the blood uric acid levels of patient 6 and patient 7 were near-normal (327 μmol/L and 378 μmol/L, respectively, as shown in Table 1; the normal range for the plasma uric acid in male children refers to the previous literature reported (Wilcox, 1996))when they were diagnosed with LND without taking uric acid-lowering drugs. The ratios of urine uric acid/creatinine were 3.0 and 2.7, respectively, which suggested increased uric acid excretion. None of the eight patients had any manifestations of gouty arthritis.

### Characteristics of Kidney Stones

**FIGURE 2:**
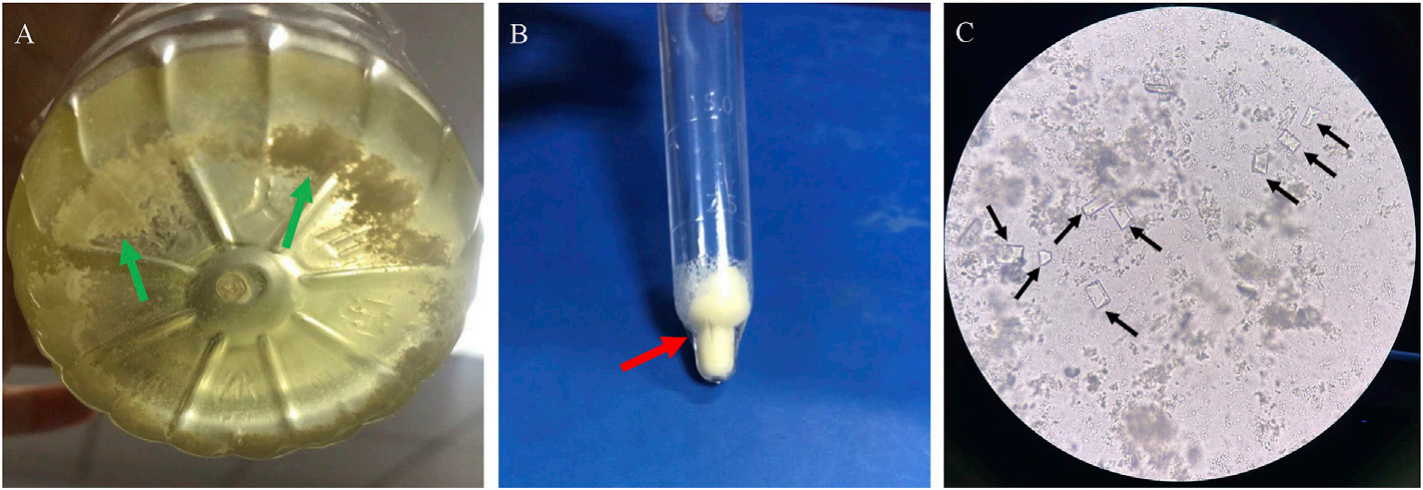
Crystalline urine in patient 8 of LND. **(A)**: Small white crystals deposited in urine **(B)**: small crystals after centrifugal precipitation, **(C)**: microscope (×200): square columnar magnesium ammonium phosphate crystal with strong refraction (black arrow).

Three patients had kidney stones or crystals (patients 1, 5, and 8) at the time of LND diagnosis. Before the diagnosis of LND in patient 1, he had a history of multiple urinary tract stones and repeated urinary tract infections, and analysis of his stone revealed that it was hydrogen urate. Although patient 5 had been taking uric acid-lowering drugs since he was 7 months old, there were still small crystals in his kidneys at the last follow-up when he was 2 years old. For patient 8, although uric acid-lowering drugs were taken in time after diagnosis, there were still small kidney stones during the 1-year follow-up. He had excreted crystalline urine twice, and microscopic examination revealed crystals of magnesium ammonium phosphate (Figure 2).

### Characteristics of Kidney Function

Patients 1 and 2 had decreased renal function at the time of diagnosis of LND. Before the diagnosis of LND, patient 1 had a history of multiple urinary tract stones and repeated urinary tract infections. He was once subjected to percutaneous nephrolithotomy due to severe renal obstruction and infection caused by stones, and analysis of the stone composition revealed that it was hydrogen urate. His urinalysis showed that the specific gravity of urine was normal, and there was no albuminuria or microalbuminuria except for a small number of white blood cells. The ultrasound (US) examination of patient 2 showed that the kidney size was normal, the renal parenchyma echo was enhanced, the cortex and pulp boundary were unclear, and no urinary calculi were found. Urinalysis showed that α1 microglobulin and β2 microglobulin were elevated, and the specific gravity of urine was normal. The parents of both patients refused further kidney pathological examination.

### Dysarthria and Dysphagia

All patients had a delay of language development and dysarthria, could only pronounce monosyllabic words and individual simple reduplicated words, and could not speak complete sentences. Usually, only their caregivers can understand their pronunciation. All eight patients had difficulty chewing and swallowing to varying degrees and could only eat a liquid diet.

### Characteristics of Drugs Taken

After diagnosis, among the six patients with a high uric acid phenotype, five patients took allopurinol (2.5–8 mg/kg.d) to control uric acid. Patient 8 took febuxostat (0.25 mg/kg.d) to control uric acid, and as of the last follow-up (3 years after taking febuxostat), no adverse reactions related to febuxostat were observed. Six patients took clonazepam (0.04–0.08 mg/kg.d) to stabilize mood and reduce muscle tension. S-Adenosylmethionine (SAMe, 23 mg/kg.d) was administered to patient 8 for more than 3 3 months, but symptoms of self-injury did not improve. Clonazepam was not observed to be helpful in reducing self-harm behavior. Patient 1 took estazolam (0.05 mg/kg.d) to control epilepsy. Most of them received sodium bicarbonate or sodium-potassium hydrogen citrate granules to alkalize the urine. During intermittent follow-up of 3 months to 3 years, there was no evidence that any of these drugs could improve dyskinesia or self-mutilation.

### Genetics

**FIGURE 3:**
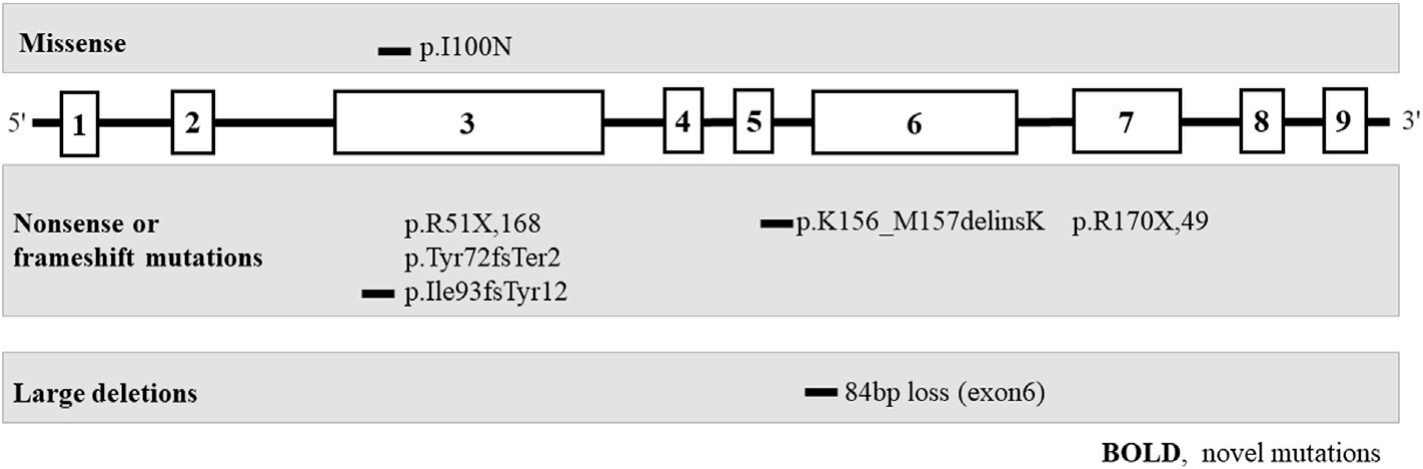
Pathogenic variants of the *HPRT1* gene (bold, novel mutation) in eight unrelated Chinese families with Lesch-Nyhan disease. Most are private genetic variants, p. R51X,168, which were confirmed in two families.

The genetic phenotypes of the eight patients are provided in Table 2; Figure 3.

**TABLE 2:** Molecular analysis of the *HPRT1* gene in 8 Chinese patients from 8 unrelated families with LND.

Patient No	Mutation type	Exon	Variants	Amino acid alteration	Source	AGMG classification
1	Missense	exon3	c.212_c.213insG	p.Tyr72fsTer2	mother	LP
2	Nonsense	exon3	c.151C > T	p.R51X,168	mother	LP
3	Nonsense	exon3	c.151C > T	p.R51X,168	mother	LP
4	Missense	**exon3**	**c.299T > A**	**p.I100N**	mother	LP
5	Deletion	**exon3**	**c.277_281delATTGC**	**p.Ile93fsTyr12**	De nove	LP
6	Deletion	**exon6**	**loss**	**84 bp**	mother	LP
7	Deletion	**exon6**	**c.468_470delGAT**	**p.Lys156_Met157delinsLys**	mother	LP
8	Nonsense	exon7	c.508C > T	p.R170X,49	mother	P

Bold, novel mutations; others represent mutations that have been reported in the Human Gene Mutation Database (URL http://www.hgmd.cf.ac.uk/ac/index.php).

Eight different pathogenic variants in HPRT1 were identified from 8 independent pedigrees, including four novel mutations [c.299 (exon 3) T > A; loss (exon: 6) 84 bp; c.277_281delATTGC; c.468_470delGAT]. There were no evident mutational hotspots. Truncating pathogenic variants, including frameshift and nonsense mutations, were the most common (5/7, 71.4%), followed by missense mutations (1/7, 14.3%) and large deletions (1/7, 14.3%). Family screening was performed in all eight patients. Pathogenic variants in seven probands (87.5%) were inherited from their mothers, and *de novo* mutations occurred in one of eight probands (12.5%). All heterozygous females were clinically normal. Variants of unknown significance identified in Patient 1–8 were listed in Supplementary Tables 1 to 8.

## Discussion

Although it was characterized 56 years ago (Lesch and Nyhan, 1964), it is still unclear how HGPRT enzyme deficiency causes such profound neurobehavioral symptoms. As mentioned above, the disease characteristics we observed are mostly consistent with the results obtained by other countries and centers (Bertelli et al., 2004; Madeo et al., 2019). However, we are shocked by the burden of disease on patients and their families, which is what we want to emphasize. The hyperuricaemia of individual patients was ignored without standardized management and treatment. Since the emergence of SIB is age-related, the diagnosis of LND is often delayed until self-mutilation becomes evident (Nyhan, 2008). As in our study, only two patients were diagnosed before self-injurious symptoms appeared. What bothers family members most is that their children are constantly hurt by self-mutilation, and there is no effective method or medicine to help them out of the predicament. Although individuals experience pain from these behaviors, they cannot stop the behavior on their own.

Harm behavior that violates personal wishes and is beyond the control of the individual is the behavioral phenotype of LND (Torres et al., 2012). Harm behavior in LND is usually grouped into four categories: (Torres and Puig, 2007): self‐inflicted harm; (Fu et al., 2014); harm/damage to other people/objects; (Nguyen et al., 2017); harm to communication in progress; and (Jinnah) harm to activity in progress (Sebesta et al., 2008). The harmful behaviors of LND patients in our study were mainly self‐inflicted harm (biting buccal mucosa, fingers, lips, and tongue). Due to the severity of SIB and its impact on quality of life, the management of SIB is necessary and challenging. Drug treatment often becomes an important part of the treatment plan. It has been reported that SAMe can improve SIBs and reduce dystonia. However, a drug clinical trial of SMAe showed that most people’s behavior deteriorates (Dolcetta et al., 2013). Our study also showed that SMAe has a poor effect on improving SIB and mood. It has also been reported that risperidone has an antagonistic effect on SIBs in patients with LND (Allen and Rice, 1996), 5-hydroxytryptophan produces a significant reduction in athetoid movement and has a sedative effect in LND (Frith et al., 1976), and ecopipam may reduce the severity of SIBs in LND (Khasnavis et al., 2016). However, there is no high-quality evidence to support any drug treatment for SIBs. In nondrug therapy, pediatricians and patients’ caregivers also supported tooth extraction as one of the important means to control SIB (James et al., 1989; Cotton et al., 2018), while dental experts preferred to rely on tooth protectors or lip protectors to reduce injuries (Limeres et al., 2013). Jinnah et al. suggested that rapid tooth extraction should be considered as part of the nursing standard when necessary (Goodman et al., 2014). However, in our study, 3 patients had permanent facial disfigurement, and none of the parents chose tooth extraction to control self-harm behavior. Parents were afraid of bleeding, infection, and anesthesia risks during tooth extraction. Perhaps the underlying reason was a cultural identity. In other words, tooth extraction is a big deal in Chinese, they think although it is only a small tooth, it is still a part of the body.

From birth, LND will produce too much uric acid, and hyperuricemia is a common phenomenon in LND (Torres et al., 2012), which is often overlooked because hyperuricemia may be mild. Rarely, the serum uric acid concentration can be normal (Sebesta et al., 2008). In our study, two patients (patient 6 and patient 7) had near-normal blood uric acid levels without taking uric acid-lowering drugs, and the ratio of urine uric acid/creatinine was 2.7 and 3.0, respectively. In infants and young children, increased renal clearance can effectively remove uric acid from the blood; therefore, there may be borderline hyperuricemia in *HPRT1* disorders (Torres et al., 2012). Some scholars suggest that the ratio of urine uric acid/creatinine can be used as a screening test for hereditary purine metabolism disorders based on the age of patients (Kaufman et al., 1968). The difference in the degree of hyperuricaemia in LND may be related to GLUT9 single nucleotide polymorphisms (SNPs) (Torres and Puig, 2018). The management of hyperuricemia in LND remains a clinical challenge. Allopurinol, as the first-line drug for the treatment of children with hyperuricemia, is most commonly used in patients with LND. However, there are no current consensus guidelines for the optimal dosage of allopurinol to avoid the risk of iatrogenic xanthine urolithiasis, and the recommended dosage of allopurinol starts at 5–10 mg/kg per day (Pais et al., 2006). Taking allopurinol (average dose 6.44 mg/kg/d) can reduce the serum uric acid concentration in LND by 50% (Torres et al., 2006). As a new specific xanthine oxidase inhibitor, febuxostat has a good effect of lowering uric acid and is especially suitable for patients with chronic renal insufficiency. Due to its high price and potential cardiovascular risk, the guidelines only recommend febuxostat as the first-line uric acid-lowering treatment drug for gout patients (Khanna et al., 20122012). However, there is insufficient evidence that febuxostat increases the risk of sudden cardiac death in Asian populations (White et al., 2018). Patient 8 had been taking febuxostat since the diagnosis of LND, his blood uric acid was well controlled, and no adverse reactions related to febuxostat were observed during the follow-up of 3 years. We found that both patients in our study who had decreased renal function at the time of diagnosis had a history of delayed diagnosis and treatment of hyperuricaemia. In previously published cases, the main cause of renal insufficiency seems to be attributed to urate nephropathy and acute obstructive nephropathy (Pela et al., 2008; Thumfart et al., 2016; Ambarsari et al., 2020). It has also been reported that pathogenic variants in the *HPRT1* gene lead to renal calcinosis and renal insufficiency (Vargiami et al., 2016). Whether there is a mechanism other than hyperuricemia in patients with renal insufficiency needs to be further observed and studied.

This is the first study to summarize the natural history of LND patients in mainland China. We describe four new mutations: c.299 (exon 3) T > A; loss (exon: 6) 84 bp; c.277_281delATTGC; and c.468_470delGAT. There were still some limitations in our study. First, functional analyses of novel mutations were not performed to confirm the results; second, HGPRT enzymatic testing was not performed to further improve the diagnosis; however, as recommended in GeneReviews: a male proband with suggestive clinical and laboratory findings, a hemizygous pathogenic variant in *HPRT1* identified by molecular genetic testing and/or low HGPRT enzyme activity can be diagnosed as *HPRT1* disorder (Jinnah). Four of the eight variants we reported have previously been confirmed to be LND-related pathogenic variants (Jinnah et al., 2000; Yamada et al., 2011). Among the four new mutations, there is one missense mutation and three deletion mutations, which change the type and sequence of polypeptide chain amino acids and affect the expression of the HGPRT protein. Moreover, the clinical characteristics of all patients are highly consistent with LND. Therefore, the detection of HGPRT enzyme activity is not necessary. However, commercialized HGPRT enzyme activity detection reagents are indeed conducive to clinicians’ screening of suspected HPRT1 diseases and early diagnosis. Finally, our study lacks data on the intelligence and cognitive function of LND patients because most of the patients show fear and insecurity when exposed to strange environments or people, which induces aggravation of self-injurious behavior and/or unstoppable crying.

Similar to other rare diseases, the management of LND involves a multidisciplinary team (neurology, rehabilitation, oral cavity, kidney, metabolism, etc.). Although there are various multidisciplinary team (MDT) teams in most third-class hospitals in China, the MDT team for LND has not been established in any hospital. Patients with LND often cannot be fully managed after diagnosis. Children with LND often become scared and anxious when in the hospital, and their behavior and neuromotor disorders will worsen, which makes it more difficult for doctors to evaluate and treat them. Therefore, we suggest that LND screening should be carried out in patients with intellectual disability, dystonic movement disorder and hyperuricemia, and the commercial HGPRT enzyme activity detection reagent can greatly improve the detection efficiency and reduce the screening cost. We believe that increasing public attention to LND with the help of social forces can improve the situation of LND patients, and increasing the publicity and popularization of LND among medical professionals can reduce missed diagnoses and misdiagnoses. The promotion of basic research, drug development (such as enzyme replacement therapy) and gene therapy research on LND will fundamentally change the long-term prognosis of children with LND.

## Conclusion

With this study, we have described the phenotypic and molecular spectrum of LND in eight Chinese families, including four novel mutations, which improve the knowledge of the natural history of the disease and expand our understanding of LND, outlining the background for future management recommendations.

## Data Availability

The datasets presented in this article are not readily available because data cannot be shared publicly because of patient confidentiality. Data are available from Children's Hospital of Zhejiang University Institutional Data Access/Ethics Committee (contact via zuchiec@163.com) for researchers who meet the criteria for access to confidential data. Requests to access the datasets should be directed to zuchiec@163.com.

## Supplementary Material

Supplementary Table S1 Variants of unknown significance identified in Patient 1.

Supplementary Table S2 Variants of unknown significance identified in Patient 2.

Supplementary Table S3 Variants of unknown significance identified in Patient 3.

Supplementary Table S4 Variants of unknown significance identified in Patient 4.

Supplementary Table S5 Variants of unknown significance identified in Patient 5.

Supplementary Table S6 Variants of unknown significance identified in Patient 6.

Supplementary Table S7 Variants of unknown significance identified in Patient 7.

Supplementary Table S8 Variants of unknown significance identified in Patient 8.

## Abbreviations

BWA, Burrows–Wheeler Aligner; ESP, Exome Sequencing Project; ExAC, Exome Aggregation Consortium; GAKT, Genome Analysis Toolkit; HGMD, Human Gene Mutation Database; HGPRT, hypoxanthine-guanine phosphoribosyltransferase; IQR, interquartile range; LND, Lesch-Nyhan disease; MDT, multidisciplinary team; MRI, magnetic resonance imaging; NCBI, National Center for Biotechnology Information; OMIM, Online Mendelian Inheritance in Man; PolyPhen-2, Polymorphism Phenotyping v2; SAMe, S-adenosylmethionine; SIB, self-injurious behavior; SIFT, Scale-invariant feature transform; SNPs, single nucleotide polymorphisms; SNPs, single nucleotide polymorphisms; US, ultrasound.
