# Supplementary material for: Description of the Molecular and Phenotypic Spectrum of Lesch-Nyhan Disease in Eight Chinese Patients
Source: Front Genet. 2022 Apr 26;13:868942. doi: 10.3389/fgene.2022.868942 (PMC9086273; doi:10.3389/fgene.2022.868942)
Supplement: Supplementary file 1 [file DataSheet1.docx]

Supplementary Table 1. Variants of unknown significance were identified in Patient 1.

| **Gene** | **Chromosome position(GRCh37/hg19)** | **cDNA** | **Zig** | **Protein** | **Disease ^1^ (OMIM: number)** | **Genetic model** | **Class ^2^ (ACMG)** | **Origin** |
| --- | --- | --- | --- | --- | --- | --- | --- | --- |
| *PRDX1* | Chr1:45977006 | c. 595A>T | het | p.K199*,1 | METHYLMALONIC ACIDURIA AND HOMOCYSTINURIA, cblC TYPE(OMIM:277400) | AR | LP | Paternal |
| *SEPN1* | Chr1:26135571 | c.802C>T | het | p.R268C | RIGID SPINE MUSCULAR DYSTROPHY 1 (OMIM:602771)  MYOPATHY, CONGENITAL, WITH FIBER-TYPE DISPROPORTION(OMIM:255310) | AR  AD/AR | LP | Maternal |

Zig: homozygosity/heterozygosity; het: heterozygote; hom: homozygotes (number of homozygotes in GnomAD database); Inh: inheritance; PP2: Polyphen2 (D - Most likely, damaging; P - Possibly damaging; B - Benign); LP: Likely pathogenic; LB: Likely Benign; US: Uncertain Significance

1. Pathologies previously associated with mutations in these genes. In brackets, OMIM number of the associated disease (according to OMIM).

2. According to the ACMG/AMP 2015 guideline

Supplementary Table 2. Variants of unknown significance were identified in Patient 2.

| **Gene** | **Chromosome position(GRCh37/hg19)** | **cDNA** | **Zig** | **Protein** | **Disease ^1^ (OMIM: number)** | **Genetic model** | **Class ^2^ (ACMG)** | **Origin** |
| --- | --- | --- | --- | --- | --- | --- | --- | --- |
| *F5* | Chr1:169524538 | c.1000A>G | het | p.R334G | THROMBOPHILIA DUE TO ACTIVATED PROTEIN C RESISTANCE(OMIM:188055) | AD | LP | Paternal |
| *SLC4A1* | Chr17:42330695 | c.2102G>A | het | p.G701D | RENAL TUBULAR ACIDOSIS, DISTAL, 4, WITH HEMOLYTIC ANEMIA (OMIM:611590) | AR | LP | Paternal |

Zig: homozygosity/heterozygosity; het: heterozygote; hom: homozygotes (number of homozygotes in GnomAD database); Inh: inheritance; PP2: Polyphen2 (D - Most likely, damaging; P - Possibly damaging; B - Benign); LP: Likely pathogenic; LB: Likely Benign; US: Uncertain Significance

1. Pathologies previously associated with mutations in these genes. In brackets, OMIM number of the associated disease (according to OMIM).

2. According to the ACMG/AMP 2015 guideline

Supplementary Table 3. Variants of unknown significance were identified in Patient 3.

| **Gene** | **Chromosome position(GRCh37/hg19)** | **cDNA** | **Zig** | **Protein** | **Disease ^1^ (OMIM: number)** | **Genetic model** | **Class ^2^ (ACMG)** | **Origin** |
| --- | --- | --- | --- | --- | --- | --- | --- | --- |
| *LRP5* | Chr11:68216447 | c.4757C>T | het | p.AI586 V | OSTEOPOROSIS-PSEUDOGLIOMA SYNDROME (OMIM:259770) | AR | LP | Maternal |
| *NBAS* | Chr2:15564561 | c.2455A>T | het | p.S819C | SHORT STATURE, OPTIC NERVE ATROPHY, AND PELGER-HUET ANOMALY (OMIM:614800) | AR | LP | Maternal |
| *RAF1* | Chr3:12626049 | c.1911C>A | het | p.C637X,12 | LEOPARD SYNDROME 2 (OMIM:611554) | AD | P | Maternal |
| *TRAPPC9* | Chr8:141321351 | c.1912G>A | het | p.V638I | MENTAL RETARDATION, AUTOSOMAL RECESSIVE 13 (OMIM:613192) | AR | LP | Paternal |

Zig: homozygosity/heterozygosity; het: heterozygote; hom: homozygotes (number of homozygotes in GnomAD database); Inh: inheritance; PP2: Polyphen2 (D - Most likely, damaging; P - Possibly damaging; B - Benign); LP: Likely pathogenic; LB: Likely Benign; US: Uncertain Significance

1. Pathologies previously associated with mutations in these genes. In brackets, OMIM number of the associated disease (according to OMIM).

2. According to the ACMG/AMP 2015 guideline

Supplementary Table 4. Variants of unknown significance were identified in Patient 4.

| **Gene** | **Chromosome position(GRCh37/hg19)** | **cDNA** | **Zig** | **Protein** | **Disease ^1^ (OMIM: number)** | **Genetic model** | **Class ^2^ (ACMG)** | **Origin** |
| --- | --- | --- | --- | --- | --- | --- | --- | --- |
| *ASS1* | Chr9:133364851 | c.970G>A | het | p.G324S | CITRULLINEMIA (OMIM:215700) | AR | LP | Paternal |

Zig: homozygosity/heterozygosity; het: heterozygote; hom: homozygotes (number of homozygotes in GnomAD database); Inh: inheritance; PP2: Polyphen2 (D - Most likely, damaging; P - Possibly damaging; B - Benign); LP: Likely pathogenic; LB: Likely Benign; US: Uncertain Significance

1. Pathologies previously associated with mutations in these genes. In brackets, OMIM number of the associated disease (according to OMIM).

2. According to the ACMG/AMP 2015 guideline

Supplementary Table 5. Variants of unknown significance were identified in Patient 5.

| **Gene** | **Chromosome position(GRCh37/hg19)** | **cDNA** | **Zig** | **Protein** | **Disease ^1^ (OMIM: number)** | **Genetic model** | **Class ^2^ (ACMG)** | **Origin** |
| --- | --- | --- | --- | --- | --- | --- | --- | --- |
| *ARSA* | Chr22:51065757 | c.302G>T | het | p.G101V | METACHROMATIC LEUKODYSTROPHY (OMIM:250100) | AR | P | Paternal |
| *ATP7B* | Chr13:52532469 | c.2333G>T | het | p.R788L | METACHROMATIC LEUKODYSTROPHY (OMIM:277900) | AR | P | Maternal |

Zig: homozygosity/heterozygosity; het: heterozygote; hom: homozygotes (number of homozygotes in GnomAD database); Inh: inheritance; PP2: Polyphen2 (D - Most likely, damaging; P - Possibly damaging; B - Benign); LP: Likely pathogenic; LB: Likely Benign; US: Uncertain Significance

1. Pathologies previously associated with mutations in these genes. In brackets, OMIM number of the associated disease (according to OMIM).

2. According to the ACMG/AMP 2015 guideline

Supplementary Table 6. Variants of unknown significance were identified in Patient 6.

| **Gene** | **Chromosome position(GRCh37/hg19)** | **cDNA** | **Zig** | **Protein** | **Disease ^1^ (OMIM: number)** | **Genetic model** | **Class ^2^ (ACMG)** | **Origin** |
| --- | --- | --- | --- | --- | --- | --- | --- | --- |
| *NALCN* | Chr13:101753130 | c.3162+5(IVS27)G>C | het | **_**  (NM_052867) | HYPOTONIA, INFANTILE, WITH PSYCHOMOTOR RETARDATION AND CHARACTERISTIC FACIES 1 (OMIM:615419) | AR | US | Maternal |
| *ABCA2* | Chr9:139909342 | c.3972+23(IVS25)G>T | het | **_**  (NM_212533) | INTELLECTUAL DEVELOPMENTAL DISORDER WITH POOR GROWTH AND WITH OR WITHOUT SEIZURES OR ATAXIA (OMIM:618808) | AR | US | Maternal |
| *SEPN1* | Chr1:26131702 | c.473T>C | het | p.I158T | RIGID SPINE MUSCULAR DYSTROPHY 1 (OMIM:602771) | AR | LP | Paternal |

Zig: homozygosity/heterozygosity; het: heterozygote; hom: homozygotes (number of homozygotes in GnomAD database); Inh: inheritance; PP2: Polyphen2 (D - Most likely, damaging; P - Possibly damaging; B - Benign); LP: Likely pathogenic; LB: Likely Benign; US: Uncertain Significance

1. Pathologies previously associated with mutations in these genes. In brackets, OMIM number of the associated disease (according to OMIM).

2. According to the ACMG/AMP 2015 guideline

Supplementary Table 7. Variants of unknown significance were identified in Patient 7.

| **Gene** | **Chromosome position(GRCh37/hg19)** | **cDNA** | **Zig** | **Protein** | **Disease ^1^ (OMIM: number)** | **Genetic model** | **Class ^2^ (ACMG)** | **Origin** |
| --- | --- | --- | --- | --- | --- | --- | --- | --- |
| *TRPV4* | Chr12:140226217 | c.2196C>G | het | p.I732 M | SCAPULOPERONEAL SPINAL MUSCULAR ATROPHY (OMIM:181405) | AD | US | Maternal |
| *ANO3* | Chr11:26547244 | c.737+15(IVS7)A>C | het | **_**  (NM_031418) | DYSTONIA 24 (OMIM:615034) | AD | US | Paternal |
| *PLEKHG2* | Chr19:39914694 | c.2924A>G | het | p.Asp974G | LEUKODYSTROPHY AND ACQUIRED MICROCEPHALY WITH OR WITHOUT DYSTONIA (OMIM:616763) | AR | US | Maternal |
| *SLC30A10* | Chr1:220101169 | c.614G>A | het | p.G205E | HYPERMANGANESEMIA WITH DYSTONIA 1 (OMIM:613280) | AR | US | Maternal |

Zig: homozygosity/heterozygosity; het: heterozygote; hom: homozygotes (number of homozygotes in GnomAD database); Inh: inheritance; PP2: Polyphen2 (D - Most likely, damaging; P - Possibly damaging; B - Benign); LP: Likely pathogenic; LB: Likely Benign; US: Uncertain Significance

1. Pathologies previously associated with mutations in these genes. In brackets, OMIM number of the associated disease (according to OMIM).

2. According to the ACMG/AMP 2015 guideline

Supplementary Table 8. Variants of unknown significance were identified in Patient 8.

| **Gene** | **Chromosome position(GRCh37/hg19)** | **cDNA** | **Zig** | **Protein** | **Disease ^1^ (OMIM: number)** | **Genetic model** | **Class ^2^ (ACMG)** | **Origin** |
| --- | --- | --- | --- | --- | --- | --- | --- | --- |
| *UMOD* | Chr16:20362132 | c.-73 (exon 2) A >T | het | **_**  (NM_003361) | TUBULOINTERSTITIAL KIDNEY DISEASE, AUTOSOMAL DOMINANT, 1 (OMIM:162000) | AD | US | Maternal |
| *GNAL* | Chr18:11876613 | c.1163-7T>G(IVS10) | hom | (NM_182978) | DYSTONIA 25 (OMIM:615073) | AD | US* | Maternal; Paternal |

Zig: homozygosity/heterozygosity; het: heterozygote; hom: homozygotes (number of homozygotes in GnomAD database); Inh: inheritance; PP2: Polyphen2 (D - Most likely, damaging; P - Possibly damaging; B - Benign); LP: Likely pathogenic; LB: Likely Benign; US: Uncertain Significance；US*: Uncertain significance, mutation is located near the splice site.

1. Pathologies previously associated with mutations in these genes. In brackets, OMIM number of the associated disease (according to OMIM).

2. According to the ACMG/AMP 2015 guideline
